# Appraisal and validation of a method used for detecting heavy metals in poultry feed in Bangladesh

**DOI:** 10.14202/vetworld.2022.2217-2223

**Published:** 2022-09-17

**Authors:** Md. Mosharaf Hossain, Abu Sayeed Md. Abdul Hannan, Md. Mostofa Kamal, Mohammad Abul Hossain, Shamshad B. Quraishi

**Affiliations:** 1 Department of Livestock Services, Quality Control Laboratory, Dhaka, Bangladesh; 2 Department of Dairy and Poultry Science, Chattogram Veterinary and Animal Sciences University, Chattogram, Bangladesh; 3 Analytical Chemistry Laboratory, Atomic Energy Centre, Bangladesh Atomic Energy Commission, Dhaka, Bangladesh

**Keywords:** graphite furnace atomic absorption spectrometry, method validation, poultry feed, toxic metals (Pb, Cr, and Cd)

## Abstract

**Background and Aim:**

Low concentrations of heavy metals are toxic and pose a serious threat to human health and the environment. Therefore, regular assessments of the toxic metal content in poultry feed are crucial for evaluating feed quality and customer safety. It is difficult to determine the heavy metals in the poultry feed at the trace amount. Therefore, this study aimed to validate this method through the detection of three heavy metals, chromium (Cr), cadmium (Cd), and lead (Pb), in poultry feed samples.

**Materials and Methods:**

Graphite furnace atomic absorption spectrometry (GF-AAS) method was used to analyze the heavy metals in poultry feed according to the guidelines given by the Council Directive 333/2007/EC, Commission Decision 657/2002/EC. In this study, various parameters such as linearity check, limit of detection (LOD), limit of quantification (LOQ), recovery percentage, precision checks, repeatability, reproducibility, and uncertainty measurement were considered to validate and assess the method following international guidelines. Heavy metals, such as Pb, Cr, and Cd, were analyzed from the feed samples in the laboratory using the GF-AAS method (Model: AA-7000 Shimadzu, Japan) with high purity argon as the inert gas, and the absorbance was read at wavelengths of 283.0, 357.9, and 228.8 nm, respectively.

**Results:**

The coefficient of variation (CV%) for system suitability and precision data was <10% for all the metals (Pb, Cr, and Cd) detected in this study. The overall CV% of repeatability and reproducibility ranged from 8.70% to 8.76% and 8.65% to 9.96%, respectively. The linearity of the calibration curves was excellent (r^2^ > 0.999) at various concentration levels for the three different metals. The recovery (%) was found to be 94.53, 93.97, and 101.63% for Pb, Cr, and Cd, respectively. The LOD values in feed were 0.065, 0.01, and 0.11 mg/kg, and the LOQ values were 0.22, 0.03, and 0.38 mg/kg for Cr, Cd, and Pb, respectively. The values recorded for the measurement uncertainty (%) were 11.48, 4.43, and 12.42% for Cr, Cd, and Pb, respectively.

**Conclusion:**

The results show that these study criteria or parameters have met the validated or acceptable range. Therefore, it is a reliable technique that can be used undoubtedly for the routine analysis of heavy metals in poultry feed samples across the globe.

## Introduction

Bangladesh is an agro-based developing country. Bangladesh’s small- and large-scale chicken farms are fast growing and supply meat, eggs, and employment opportunities. More than 50% of this country’s population is involved in agricultural and livestock farming. In a developing nation like Bangladesh, the poultry industry provides both direct and indirect employment opportunities and is a vital part of agricultural farming systems [1]. Both developed and developing countries face an alarming problem of environmental pollution with heavy metals and metalloids [2]. Heavy metals are important and have been extensively studied for their detrimental effects and bioaccumulation in food chains or feeding systems [3]. Feeding poultry requires many different feedstuffs to balance the ration. Most of these feedstuffs are mainly derived from plants grown in the soil. The feedstuffs might be adulterated and contaminated differently from growing to supplying the birds. For example, fish meal collected from contaminated water can transfer heavy metals to poultry feed, as we know that the poultry diet is composed of various raw materials [4]. Feeding feed or food is a very important element for all living beings because it contains all essential nutrients for the proper nourishment of the body. All categories of people use poultry meat and egg as one of the best food items, growing rapidly worldwide. It is obvious that the rapid growth of poultry depends on the feeds and that feeds contain different pollutants, including heavy metals. These metals spread widely and release toxic substances into the environment due to natural and anthropogenic activities [5]. As a result, environmental pollution is increasing alarmingly to heavy metal toxicity, posing a great threat to human health [6]. Some heavy metals, notably cadmium (Cd), chromium (Cr), lead (Pb), and arsenic (As), are reported detrimental at low concentrations. According to the United States Environmental Protection Agency [7], metals (e.g., Cr, As, Cd, and Pb) are considered the most toxic elements for the environment. These are commonly available heavy metals that remain intact, are not degraded easily, and cause environmental and food contamination. The metals, in turn, trigger many acute or chronic disorders in humans due to their gradual accumulation in various animal body organs for a longer period [8].

In view of the above, it is obvious that poultry feeds could contain toxic heavy metals at a sufficient level and their exposure might occur after the birds consume these feed materials. The data on metal contamination or detection in poultry feedstuffs are insufficient in our country. Although there are many methods for quantifying heavy metals, further analytical laboratory results may be necessary for official control and quality assurance. For this reason, global demand is growing to develop and establish new modern laboratory techniques for providing accurate and reliable data or measurements with low concentration levels [9]. According to Food and Agricultural Organization [10], sustainable agricultural development relies on feed quality, which is considered one of Bangladesh’s main barriers to improving livestock.

For this reason, a method of graphite furnace atomic absorption spectrometry (GF-AAS) has been developed to detect toxic elements, mainly Pb, Cd, and Cr, in poultry feed samples. This method appears reliable, precise, accurate, sensitive, selective, and economical [11]. The poultry feed quality can be assessed regularly if the method is implemented and validated globally. Hence, the developed method will help to identify the toxic elements, which has a great application in laboratory examination of poultry feed samples [12]. In addition, this study would greatly benefit the poultry industry, feed miller companies, and poultry integrators.

There are several other analytical techniques available to detect toxic metals reported by previous researchers; these include flame AAS, GF-AAS, and inductively coupled plasma mass spectrometry, which are being used for the quantification of toxic metals in feedstuffs [13–15]. This study aimed to analyze the toxic metals (e.g., Pb, Cd, and Cr) in the poultry feed usually manufactured in the different feed mills of Bangladesh.

## Materials and Methods

### Ethical approval

No ethical approval is needed for conducting this sort of study, as it did not deal with any animals or living beings.

### Study period and location

The study was conducted from November 2020 to October 2021 at the Quality Control Laboratory, Department of Livestock Services, Savar, Dhaka, Bangladesh.

### Tools, chemicals, and reagents

An atomic absorption spectrophotometer (Model: AA-7000, Shimadzu, Japan) equipped with a GF (GFA 7000) and an autosampler (ASC 7000) was used. A cathode lamp was used and operated according to the manufacturer’s instructions for the analyses of Pb (283.0 nm, 10 mA, and slit 0.7 nm), Cd (228.8 nm, 10 mA, and slit 0.7 nm), and Cr (357.9 nm, 10 mA, and slit 0.7 nm), respectively. Nitric acid (HNO_3_, 69%), hydrogen peroxide (H_2_O_2_, 30%), and standards of Pb, Cd, and Cr were used for sample preparation. A microwave acid digestion system (MADS) (Ethos Easy Milestone) was used for conducting the digestion process. The standard bulk solutions were made daily by diluting respective metals stock standard solutions using 1% (w/w) (HNO_3_, 69%). The autosampler was used for preparing standard solutions. Deionized water (18 MW/cm) produced using an E-pure system (Thermo Scientific, United States) was used to prepare all the solutions and clean and wash all containers and glassware before use.

### Sample preparation and analytical procedure

Poultry feed was prepared with the conventional feed ingredients locally available in Bangladesh following the standard prescribed by the respective breeder company or National Research Council [16], as shown in Table-1. The samples were then dried and ground by mortar and pestle and put in an air sealable bag before laboratory analysis. For the digestion of samples, approximately 0.5 g of feed sample was weighed and digested with 8 mL of HNO_3 (_69%) and 2 mL of H_2_O_2 (_30%) in acid-prewashed Teflon vessels. A MADS (Ethos Easy Milestone) was used for the digestion procedure. After digestion, the sample was diluted with 50 mL final volume with deionized water. The reference material analytical blanks were also prepared with each batch of digestion sets. All samples were prepared in triplicate. Diluted samples and the standard solution were separately put into a set of fresh tubes for atomic absorption spectrophotometer (AAS Shimadzu AA-7000). Final heavy metals, Pb, Cr, and Cd, were measured at 283.0, 357.9, and 228.8 nm, respectively.

**Table-1:** Composition of broiler grower feed.

Ingredients	Amount (%)
Corn/maize	59.00
Wheat	3.32
Soybean meal (46%)	23.00
Corn gluten (60%)	6.40
Palm oil	4.20
Dicalcium phosphate	1.70
Limestone	1.40
Table salt (NaCl)	0.25
Choline chloride	0.06
Vitamin mineral premix	0.15
L-lysine	0.30
DL-methionine	0.22
Nutrient	
ME	3200 Kcal/kg
CP	20.0%

DL = Dextrorotation and levorotation, ME = Metabolizable energy, CP = Crude protein.

### Preparation of spiking solution for Cd, Cr, and Pb

About 0.5 g (500 mg) sample was obtained from the digestion vessel, and 8 mL HNO_3 (_69%), 2 mL H_2_O_2 (_30%), and 0.5 mL (500 uL) of each 1000 ppb of Cd, Cr, and Pb solutions were added before the solution was mixed and left to digest. Finally, the sample was prepared with water up to 50 mL, and 5 mL of this solution was then diluted to 10 mL with a solution of 1% nitric acid.

### Criteria for the validation of the proposed method

The parameters or criteria for the validation of the proposed method in this study followed were linear range, limits for detection and quantification, accuracy percentage, precision checks, and degree of uncertainty measurement [17–19]. The validation criteria were assessed following suggested instructions or guidelines of the International Conference on Harmonization [20] and the United States Food and Drug Administration [21].

#### Linearity

Standard mixtures of Pb, Cr, and Cd were prepared and a linear equation was established for each metal by plotting the absorbance versus the concentrations to measure linearity. Three calibration curves were obtained on 3 consecutive days with a specified standard concentration of each metal. Linearity was calculated by running aqueous standard solution of each metal at final concentrations of 0.25, 0.50, 1.0, 2.0, and 4 μg/mL for Cd; 2, 4, 8, and 16 μg/mL for Cr; and 2, 5, 10, 15, 20, and 40 μg/mL for Pb. The slope, intercept, and regression coefficient (r^2^) values were retrieved from the linear regression and correlation method.

#### Recovery percentage

Three sets of spike samples (2.5, 5.0, and 7.5 mg/kg) were prepared, and each set was replicated seven times. Sample reading was taken by measuring two times. For the recovery estimation, an accurate amount of each metal’s three concentration levels (2.5, 5.0, and 7.5 mg/kg for Cr and Pb and 0.25, 0.5, and 0.75 mg/kg for Cd) was added to approximately 1.0 g of blank matrix powder. The powder was then extracted and analyzed for recovery using the formula: Recovery (%) = (amount obtained/amount spiked) × 100.

### Limit of detection (LOD), limit of quantification (LOQ), instrument quantification limit (IQL), and instrument detection limit (IDL)

The lowest qualitative and quantitative concentrations for the tested linearity range were calculated for each metal according to the guidelines of ICH2000. LOD and LOQ were calculated using the expression: k × S.D/b, where k = 3.3 for the LOD and 10 for the LOQ, SD is the standard deviation of the intercept, and b is the slope of the calibration curve tested for linearity. IDL and IQL are calculated using following formula: IDL = 3s and IQL = 10s; where, s = standards deviation.

#### Repeatability and reproducibility precision check

The precision of the method was evaluated based on repeatability and intermediate precision. The repeatability was calculated on the results obtained from the same day for seven independent mixer solutions of the variable concentration, and the intermediate precision was evaluated by calculating the repeatability of the similar concentration by two analysts on different days. The percentage of relative standard deviation was calculated to estimate the precision of this study. For reproducibility precision check, three sets of samples were prepared again after 15 days by the same analyst and after 18 days with different analysts spiking at 0.25, 0.5, and 0.75 and 2.5, 5.0, and 7.5 level and analyzed as before. The mean concentration, standard deviation, and coefficient of variation (CV%) of each level of fortified samples have been calculated.

### Statistical analysis

Raw data were inserted into a Microsoft Excel (Microsoft Office, USA) sheet for statistical analysis. The data were statistically analyzed using a Minitab statistical software version 16 [22]. The means and standard deviations of the metal concentrations in samples were calculated. Descriptive analysis was performed using different variables’ percentages, the mean, and standard deviation. Finally, a one-way analysis of variance was used to compare the level of heavy metal residues in poultry feed.

## Results

### Linearity

Table-2 shows the linear regression data of heavy metals (Pb, Cr, and Cd) found in poultry feed samples. It is obvious from the data that the values of the r^2^ were 0.999, 0.9996, and 0.9998 for Pb, Cd, and Cr, respectively.

**Table-2:** Linear regression data of heavy metals in poultry feed.

Metal	Linear range (μg/L)	Calibration with an aqueous standard solution		

		Slope±SD (µg/L)	Intercept±SD	Regression coefficient (r^2^)
Pb	2–40	0.0101±0.0006	0.0193±0.0125	0.9996
Cr	2–16	0.0309±0.0018	0.1396±0.0559	0.9998
Cd	0.25–4.0	0.2332±0.0055	0.5036±0.0911	0.9996

SD=Standard deviation, Cr=Chromium, Cd=Cadmium, Pb=Lead

### Accuracy or recovery (%) of heavy metals in poultry feed

The overall recovery % for Pb, Cr, and Cd in poultry feed samples was 94.63%, 93.97%, and 101.63%, respectively (Table-3).

**Table-3:** The recovery (%) of heavy metals in poultry feed.

Metal	Spiked analyte concentration (mg/L)	Calculated analyte concentration (n = 7) (mg/kg)	Recovery (%) (n = 7)	Overall recovery % (n = 21)
Pb	2.5	2.17	86.97	94.63
	5	4.6	91.90	
	7.5	7.87	104.93	
Cr	2.5	2.48	102.01	93.97
	5	4.50	89.92	
	7.5	6.97	92.88	
Cd	0.25	0.27	109.64	101.63
	0.50	0.44	88.84	
	0.75	0.80	106.42	

Cr=Chromium, Cd=Cadmium, Pb=Lead

### Determination of IDL, IQL, LOD, and LOQ of toxic metals in poultry feed

The values of IDL for Cr, Cd, and Pb were 0.65, 0.09, and 1.10 mg/L, whereas the values of IQL were 2.16, 0.29, and 3.68 mg/L, respectively. The results of method LOD were 0.065, 0.01, and 0.11 mg/kg, and the values for LOQ were 0.22, 0.03, and 0.38 mg/kg in the metals of Cr, Cd, and Pb, respectively (Table-4).

**Table-4:** The results of IDL, IQL, and method LoD and LoQ of the heavy metals in poultry feed.

Parameter	Heavy metal			Average

	Cr	Cd	Pb	
Instrumental IDL (μg/L)	0.65	0.09	1.10	0.61
Instrumental IQL (μg/L)	2.16	0.29	3.68	2.04
Method LoD (μg/kg)	0.065	0.01	0.11	0.061
Method LoQ (μg/kg)	0.22	0.03	0.38	0.21

IDL=Instrument detection limit, IQL=Instrument quantification limit, LoD=Limit of detection, LoQ=Limit of quantification, Cr=Chromium, Cd=Cadmium, Pb=Lead

### Repeatability and reproducibility data of heavy metals in poultry feed

The precision studies are performed by measuring the repeatability and reproducibility data, as shown in Tables-5 and 6. The data showed that the values of repeatability of CV% were 8.70%, 8.76%, and 8.75% for the Pb, Cr, and Cd, respectively (Table-5). The reproducibility data of CV% were 8.86%, 9.96%, and 8.65% for Pb, Cr, and Cd, respectively (Table-6).

**Table-5:** The repeatability data of heavy metals in poultry feed.

Metal	Day	Spike concentration (mg/kg)	Overall mean concentration (n = 21) (mg/kg)	SD	CV%	Overall CV (%)
Pb	1–3	2.50	2.33	0.21	9.07	8.70
	1–3	5.00	4.78	0.46	9.61	
	1–3	7.50	7.22	0.54	7.42	
Cr	1–3	2.50	2.58	0.32	12.39	8.76
	1–3	5.00	4.58	0.42	9.27	
	1–3	7.50	6.91	0.32	4.61	
Cd	1–3	0.25	0.26	0.02	8.70	8.75
	1–3	0.50	0.46	0.05	10.96	
	1–3	0.75	0.78	0.05	6.62	

SD=Standard deviation, CV%=Coefficient of variation, Cr=Chromium, Cd=Cadmium, Pb=Lead

**Table-6:** The reproducibility data of heavy metals in poultry feed.

Metal	Day	Spike concentration (mg/kg)	Overall mean concentration (n = 21) (mg/kg)	SD	CV%	Overall CV%
Pb	1–3	2.50	2.48	0.26	10.45	8.86
	1–3	5.00	4.81	0.45	9.27	
	1–3	7.50	7.57	0.52	6.85	
Cr	1–3	2.50	2.54	0.32	9.83	9.96
	1–3	5.00	4.63	0.48	9.04	
	1–3	7.50	6.96	0.84	11.01	
Cd	1–3	0.25	0.26	0.02	7.52	8.65
	1–3	0.50	0.49	0.06	11.37	
	1–3	0.75	0.79	0.06	7.07	

SD=Standard deviation, CV%=Coefficient of variation, Cr=Chromium, Cd=Cadmium, Pb=Lead

### Measurement of uncertainty (%) of heavy metals in the poultry feed

**Figure-1:**
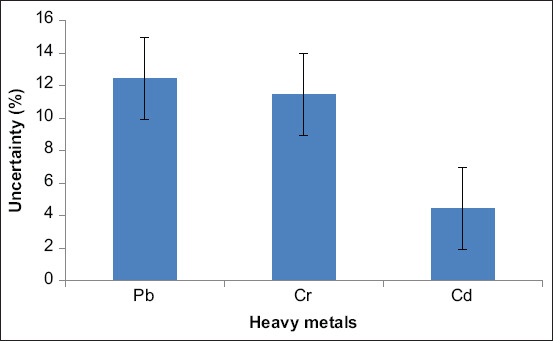
Uncertainty percentage (mean ± SE) of measured heavy metals in poultry feed.

The values of measuring uncertainty (MU) for Pb, Cr, and Cd were 12.42%, 11.48%, and 4.43%, respectively, which were shown through a graph (Figure-1). The highest uncertainty (12.42%) was observed for Pb, whereas the lowest value (4.43%) was found in Cd. Lower uncertainty values characterize higher accuracy.

## Discussion

The linearity data obtained from this study showed that it had met the acceptance level, that is, r^2^ = 0.995. The data indicate that the r^2^ value (0.999) found in this study is greater than the acceptable level (0.995), and it implies that the result is very good as the linear relationship found in this study is precise and authentic [23]. Our result is consistent with the report of previous investigators who found similar results for the validation of heavy metals in milk and fish feed samples [9, 24].

The overall recovery % was 92.63–101.63% in the detected metals in this study. The acceptable level for the recovery % ranges from –20% to +10%. Based on this fact, we can say that our analytical value of recovery % has met the standard level or criteria. Recovery (%) refers to how much extraction can be obtained by the analytical process while conducting sample testing in the laboratory [21]. Although no verified data or reference materials for poultry feed samples are available, the recovery % was done to justify the method’s accuracy [23].

LOD and LOQ are measured to detect the performance or efficacy of an instrument or an analytical method. Our results clearly show that the LODs and LOQs for the identified metals in feed samples were determined to be lower than the highest range as given by Regulations (EC) No. 1275/[25], and it has matched well according to Regulation 333/2007/EC [17].

The CV% of repeatability and reproducibility data was <10% in our analytical values of the study. The acceptable value for these parameters is considered 10% [26]. Therefore, it can be stated that the method exhibited good repeatability and reproducibility precision based on the values obtained in this study. The analytical precision was established in accordance with EC commission regulation [27]. CV% normally assesses the precision, which denotes the proximity of compromising data [28]. Measuring uncertainty (MU) calculation is necessary for every measurement according to ISO/IEC 17025 [18] because it checks the errors and omissions that occurred while conducting any assay. The reproducibility data can be used to estimate MU [29, 30]. However, despite the uncertainty or limitations, the method we developed here for detecting toxic metals in the feed samples is very simple, precise, accurate, reliable, and cost-effective as well. Thus, the proposed method can be used for routine analyses or simultaneous detection and quantification of heavy metals, which has a great implication for laboratory examination of poultry feed samples across the globe [12]. Our results are also consistent with the previous investigators. They stated similar types of remarks regarding method validation for the fish feed [9], milk [24], and detection and assessment of heavy metals in poultry foodstuffs [12, 31–35].

## Conclusion

It can be concluded that all the criteria for method validation (e.g., linearity, precision, recovery percentage, LOD and LOQ, repeatability, reproducibility, and uncertainty measurement) had been satisfied. We think that our developed method to detect heavy metals from poultry feed is very simple, easy to conduct, reliable, and cost-effective compared to other methods.

## Authors’ Contributions

MMK and SBQ: Planned and designed the study. MMH and ASMAH: Carried out the sample preparation and laboratory analysis. MMH and MAH: Statistical analyses, tabulation, interpretation, compilation, revision, edition, validation, and manuscript preparation. All authors have read and approved the final manuscript.
